# Genetic changes caused by restocking and hydroelectric dams in demographically bottlenecked brown trout in a transnational subarctic riverine system

**DOI:** 10.1002/ece3.5191

**Published:** 2019-04-29

**Authors:** Cornelya F. C. Klütsch, Simo N. Maduna, Natalia Polikarpova, Kristin Forfang, Paul Eric Aspholm, Tommi Nyman, Hans Geir Eiken, Per‐Arne Amundsen, Snorre B. Hagen

**Affiliations:** ^1^ Norwegian Institute of Bioeconomy Research (NIBIO) Svanvik Norway; ^2^ Pasvik Strict Nature Reserve Rajakoski, Murmansk Russia; ^3^ Department of Arctic and Marine Biology, Faculty of Biosciences, Fisheries and Economics UiT The Arctic University of Norway Tromsø Norway

**Keywords:** fish stocking, genetic diversity, genetic erosion, genetic integrity, habitat fragmentation, *Salmo trutta*

## Abstract

Habitat discontinuity, anthropogenic disturbance, and overharvesting have led to population fragmentation and decline worldwide. Preservation of remaining natural genetic diversity is crucial to avoid continued genetic erosion. Brown trout (*Salmo trutta* L.) is an ideal model species for studying anthropogenic influences on genetic integrity, as it has experienced significant genetic alterations throughout its natural distribution range due to habitat fragmentation, overexploitation, translocations, and stocking. The Pasvik River is a subarctic riverine system shared between Norway, Russia, and Finland, subdivided by seven hydroelectric power dams that destroyed about 70% of natural spawning and nursing areas. Stocking is applied in certain river parts to support the natural brown trout population. Adjacent river segments with different management strategies (stocked vs. not stocked) facilitated the simultaneous assessment of genetic impacts of dams and stocking based on analyses of 16 short tandem repeat loci. Dams were expected to increase genetic differentiation between and reduce genetic diversity within river sections. Contrastingly, stocking was predicted to promote genetic homogenization and diversity, but also potentially lead to loss of private alleles and to genetic erosion. Our results showed comparatively low heterozygosity and clear genetic differentiation between adjacent sections in nonstocked river parts, indicating that dams prevent migration and contribute to genetic isolation and loss of genetic diversity. Furthermore, genetic differentiation was low and heterozygosity relatively high across stocked sections. However, in stocked river sections, we found signatures of recent bottlenecks and reductions in private alleles, indicating that only a subset of individuals contributes to reproduction, potentially leading to divergence away from the natural genetic state. Taken together, these results indicate that stocking counteracts the negative fragmentation effects of dams, but also that stocking practices should be planned carefully in order to ensure long‐term preservation of natural genetic diversity and integrity in brown trout and other species in regulated river systems.

## 1 INTRODUCTION

Long‐term persistence of natural populations depends on a complex interplay of ecoevolutionary forces affecting genetic diversity and local adaptation to environmental conditions (Bijlsma & Loeschcke, 2012; Mimura et al., 2017). Habitat destruction and overexploitation of species may lead to isolated and small populations with declining effective population size and genetic diversity, and increasing genetic drift (Bijlsma & Loeschcke, 2012; Mimura et al., 2017; Piccolo, Unfer, & Lobón‐Cerviá, 2017; Vøllestad, 2017). Introduction of foreign genetic material by immigration or the release of translocated and/or hatchery‐reared individuals into the wild may replace gene pools (Laikre, Schwartz, Waples, & Ryman, 2010; Quiñones, Johnson, & Moyle, 2014). Conservation and management actions commonly aim to increase connectivity and demographic robustness by, for example, establishing dispersal corridors or releasing individuals into the wild to support local populations (Quiñones et al., 2014). However, potential genetic effects of such mitigating actions are often not considered or monitored, which increases the risk of losing natural genetic diversity in disturbed and altered populations (Araguas et al., 2009, 2017). Therefore, assessments of genetic diversity, connectivity, and structure are essential for ensuring long‐term benefits of management actions (Quiñones et al., 2014).

The linear nature of riverine ecosystems makes fragmentation a major threat to many aquatic species, which are often unable to bypass artificial barriers (Fagan, 2002; Kraabøl, Johnsen, Museth, & Sandlund, 2009; Rolls, Stewart‐Koster, Ellison, Faggotter, & Roberts, 2014). Man‐made barriers (e.g., hydroelectric power plant dams and weirs) have been shown to decrease genetic diversity by genetic drift and to increase population‐genetic differentiation in migratory fish species, including salmon, brown trout, and grayling (Gouskov, Reyes, Wirthner‐Bitterlin, & Vorburger, 2016; Heggenes & Røed, 2006; Horreo et al., 2011; Meldgaard, Nielsen, & Loeschcke, 2003; Stelkens, Jaffuel, Escher, & Wedekind, 2012). The effects caused by population fragmentation may be magnified by simultaneous destruction of important spawning grounds and nursery areas, leading to additional reductions in both natural recruitment and population size (Vøllestad, 2017).

Measures against the negative consequences of anthropogenic barriers in riverine systems include construction of fish passes (Gouskov et al., 2016; Kraabøl et al., 2009; Rolls et al., 2014), introduction of non‐native species (Caudron, Champigneulle, Vigier, Hamelet, & Guyomard, 2012; Fernández‐Cebrián, Araguas, Sanz, & Garcia‐Marin, 2014), release of hatchery‐reared individuals (Fabiani et al., 2018; Hansen, Fraser, Meier, & Mensberg, 2009; Petereit et al., 2018; Quiñones et al., 2014; Thaulow, Borgstrom, & Heun, 2013; Vøllestad & Hesthagen, 2001), and supplementary stocking with local specimens. However, fish passes have generally been found to be insufficient to prevent population subdivision even in strongly migratory species like salmonids (Noonan, Grant, & Jackson, 2012). Similarly, the introduction of non‐native species is problematic as local adaptation patterns might be disrupted (Bourret, O'Reilly, Carr, Berg, & Bernatchez, 2011; Hutchings, 2014; Jonsson & Jonsson, 2016). Consequently, supplementary stocking with regional specimens has become a widely applied alternative aiming to both maintain natural genetic diversity and integrity and counteract negative effects stemming from overexploitation, pollution, and artificial migration barriers (Saint‐Pé et al., 2018; Vøllestad & Hesthagen, 2001). However, there is mounting evidence that partial or full replacement of wild gene pools by stocked fish (Hansen et al., 2009; Laikre et al., 2010; Quiñones et al., 2014) and reduced genetic diversity and fitness in released fish (Araki, Cooper, & Blouin, 2009; Eldridge, Myers, & Naish, 2009) may compromise long‐term preservation goals. In some cases, stocking has reduced genetic differentiation across wild populations (Eldridge et al., 2009; Eldridge & Naish, 2007; Hansen et al., 2009; Kohout, Jašková, Papoušek, Šedivá, & Šlechta, 2012; Marie, Bernatchez, & Garant, 2010) and altered dispersal behavior of admixed offspring (Saint‐Pé et al., 2018). These findings have raised concerns about the loss of local adaptation (Bourret et al., 2011; Hutchings, 2014; Jonsson & Jonsson, 2016) and changes in ecologically important traits through hybridization between wild and captivity‐bred fish (Saint‐Pé et al., 2018).

The Eurasian brown trout (*Salmo trutta* L. 1758, Figure 1) is a socioeconomically important freshwater fish that is widespread in the Northern Hemisphere (Jonsson & Jonsson, 2006; Laikre, 1999; Vøllestad, 2017). It is ecologically and morphologically variable including, resident and migratory life‐history forms like anadromous (i.e., natal rivers–sea–natal rivers' migrations), as well as potamodromous (i.e., natal rivers–lakes–natal rivers' migrations) that can coexist in the same habitat. Although not endangered as such, natural brown trout populations with high genetic integrity are becoming increasingly rare across the distribution range (Araguas et al., 2009, 2017; Baric et al., 2010) due to anthropogenic habitat destruction and the long‐term practice of translocations and stocking. Hydropower developments are widespread in many countries and affect a wide range of formerly continuous brown trout populations (Heggenes & Røed, 2006; Vøllestad & Hesthagen, 2001). Therefore, supportive breeding has been widely applied to support local populations that are under pressure of fragmentation effects of dams and/or overfishing (Vøllestad & Hesthagen, 2001).

**Figure 1:**
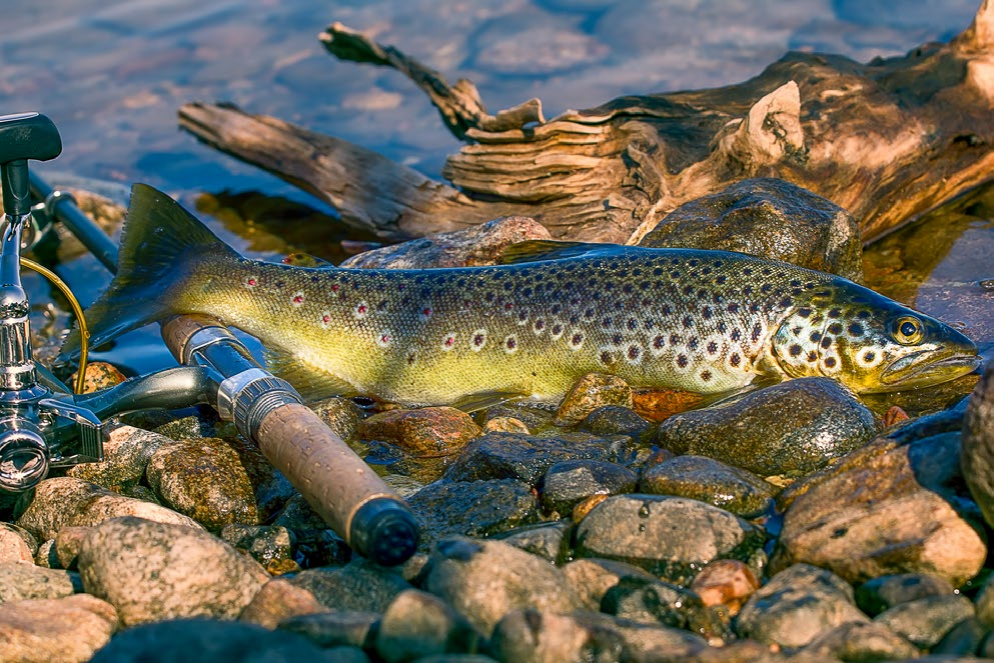
A brown trout specimen from the Pasvik River (Photo: Valery Buzun)

**Figure 2:**
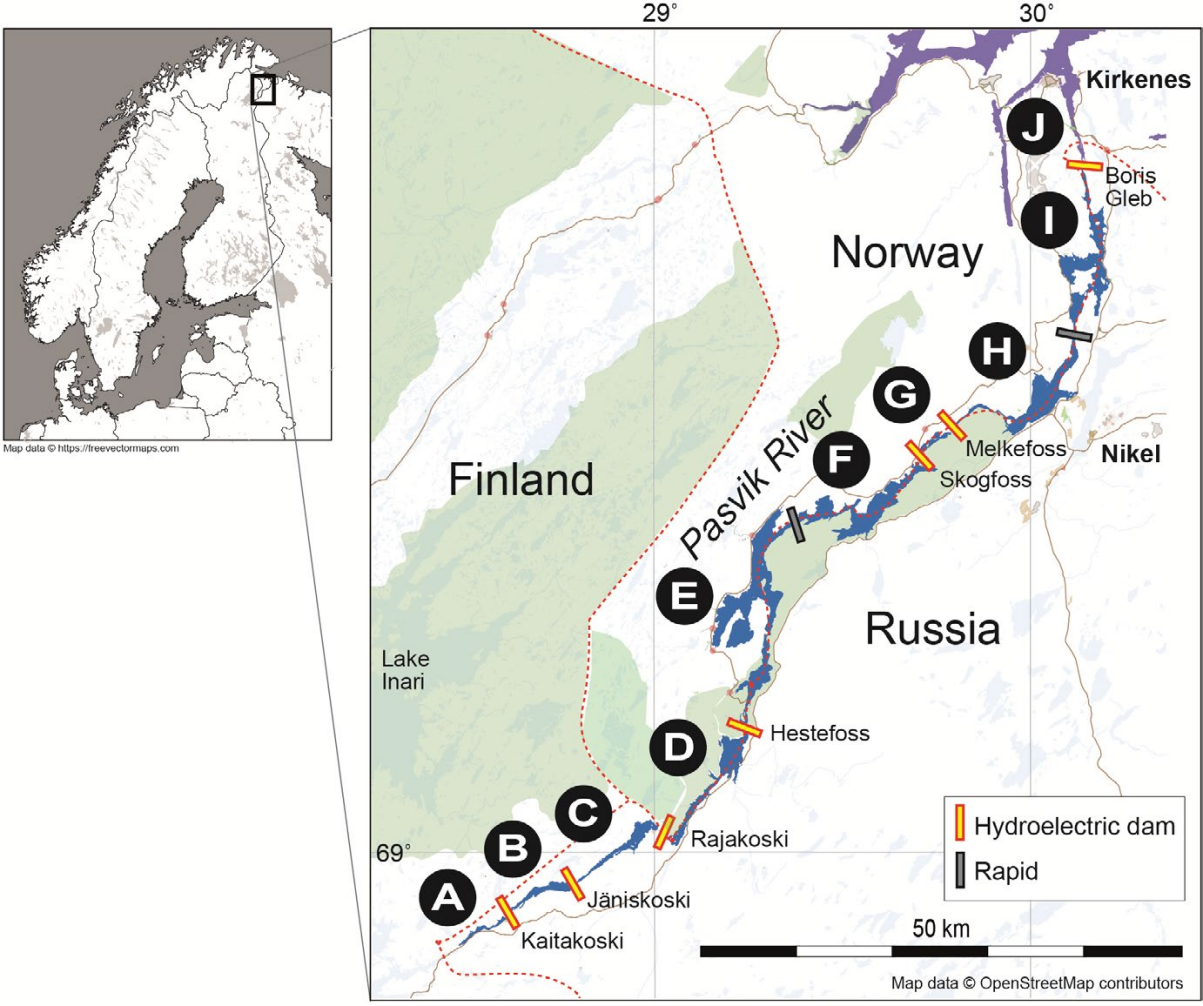
Study area showing the locations of dams and rapids in the Pasvik River. Letters denote river sections referred to in the text

Targeted studies of how anthropogenic disturbances, such as hydropower developments, and mitigating actions, such as stocking, work in concert to affect the genetic integrity of natural populations are warranted to support long‐term conservation goals in brown trout and other species facing similar challenges (Araguas et al., 2009, 2017; Baric et al., 2010; Berrebi et al., 2019; Piccolo et al., 2017). In this study, we used brown trout as a model species and compared genetic diversity and differentiation patterns along a transnational subarctic riverine system that has been partitioned by hydroelectric power dams, and that additionally is regularly stocked with fish in parts of the river system but not in others. The Pasvik River, shared between Norway, Russia, and Finland, and for a large part constituting the border between Norway and Russia, is one of the largest and most species‐rich subarctic river systems in northwestern Eurasia, and is described in sport fishing literature as one of Norway's best trout fishing destinations. However, the construction of seven hydroelectric dams from 1932 to 1978 hindered trout migration (Arnesen, 1987) and led to the destruction of many natural spawning and nursery areas (Amundsen et al., 1999; Jensen, Bøhn, Amundsen, & Aspholm, 2004). To strengthen the breeding population, approx. 5,000 offspring of local specimens are released annually between dams in the Norwegian–Russian parts of the river (Amundsen et al., 1999; Jensen et al., 2004; Table 1). Each year new parents are caught, but from the same location (i.e., zone H; Figure 2). Surveys show that 70%–90% of the trout caught in the Norwegian–Russian part of the river are stocked fish (Haugland, 2014). By contrast, no stocking of brown trout takes place in the upper Russian sections of the river, where the natural trout population is presumably more intact. Due to the dams, there is assumedly little or no gene flow between the stocked (Norwegian–Russian) and nonstocked (Russian) river sections, creating an opportunity to test hypotheses about the combined and separate genetic effects of stocking and dispersal barriers in a fine‐scale spatial context.

**Table 1:** Dam building and river information

Hydroelectric power plant	Opening date	Additional information	Between sections (Figure 1)
Kaitakoski	1959		A–B
Jäniskoski	1950	Built from 1932 to 1942; destroyed in 1944 and subsequently rebuilt	B–C
Rajakoski	1956		C–D
Hestefoss	1970	Built between 1956 and 1970	D–E
Skogfoss	1964		F–G
Melkefoss	1978		G–H
Boris Gleb	1964	Built between 1960 and 1964	I–J

This setting allowed the investigation of whether or not (a) artificial barriers reduce population‐genetic diversity within and increase genetic differentiation among subpopulations, (b) stocking increases genetic diversity within and admixture among subpopulations, (c) stocking ensures long‐term preservation of natural genetic diversity and integrity, and (d) stocking causes genetic erosion and divergence from natural state.

## 2 MATERIAL AND METHODS

### 2.1 Study system and sampling

The study area comprises the Pasvik River, which originates in Lake Inari in Finland and runs 147 km in a northeastward direction through Russia and Norway to the Arctic Ocean. The upper part of the river is located in Russia (sections A–C; Figure 2) whereas lower parts flow along the Norwegian–Russian border (sections D–J). Seven hydroelectric power plant dams, built from 1932 to 1978, partitioned the river into eight sections (Figure 2, Table 1). None of the dams have fish passes facilitating migration between river sections. Additionally, two more sections were introduced and sampled (F and I, Figure 2) that are separated by rapids from adjacent sections, but not by dams, and that mark a significant change in habitat to allow for comparative fine‐scale genetic analyses. Regular stocking in the Norwegian‐Russian river part has occurred in fairly equal numbers in sections E/F and H/I with about 50% of the stocking pool being released in these river parts. Section G has been stocked in the past with thousands of hatchery‐bred individuals, but it seems that no stocking has been conducted in this section for more than a decade. New parental fish for hatchery breeding are caught every year in zone H of the Pasvik River (Figure 2) and offspring from those breeding events are annually released afterward.

Sample collection aimed to systematically retrieve samples from all sections; however, from a few sections only very few samples could be obtained (A, *N* = 2; D, *N* = 2; and J, *N* = 1, Figure 2), so these sections were mostly excluded from statistical analyses of genetic variation and differentiation. Tissue samples (*N* = 175) consisted of adipose fin clips collected opportunistically by anglers or collected during field trips and stored in 96% ethanol until laboratory analysis.

### 2.2 Development of multiplex PCRs

In total, 40 previously described microsatellite or short tandem repeat (STR) loci were tested in the development of multiplex PCR for genotyping of brown trout. When needed, original primers were redesigned by adding PIG‐tails (Brownstein, Carpten, & Smith, 1996) and changing both amplicon lengths and annealing temperatures to provide adequate peak separation and minimize noise on the genetic analyzer (Applied Biosystems 3730xl) used in this study. The STR markers were tested first on a small sample set (*N* = 7), and later the novel multiplexes were validated on a larger sample set (*N* = 75) consisting of fish collected in the field (sections F and G; Figure 2). First, STR markers were run singly (0.5 µM primer concentration, PCR program as described under DNA extraction and multiplex PCR‐STR analysis—58°C annealing) to exclude those that were difficult to score and those that were in physical linkage (Gharbi et al., 2006). Second, the STR markers were combined in multiplexes based on fragment length, annealing temperatures, and compatibility in multiplex amplifications. Finally, PCR conditions (i.e., primer concentrations, fluorescent labeling, and annealing temperatures) were optimized to yield proportionately equal heights of fragments in the spectra. MICRO‐CHECKER 2.2.3 (Van Oosterhout, Hutchinson, Wills, & Shipley, 2004) was used to check for the presence of null alleles and scoring errors, and markers showing signs of this were excluded.

### 2.3 DNA extraction and multiplex PCR‐STR analysis

Genomic DNA was extracted from the samples using a DNeasy Blood & Tissue kit (Qiagen) and genotyped at 16 STR loci (Table 2). PCR amplifications were performed in 10 µl reactions, each containing 5.0 µl 2× Multiplex PCR Master Mix (Qiagen), 1.0 µl 10× primer mix, 0.05 µl BSA, 1.0 µl DNA template, and 2.95 µl RNase‐free water. The PCR cycling profile consisted of an initial denaturation step at 95°C for 10 min, followed by 28 cycles including a denaturation step at 94°C for 30 s, annealing at 55°C/58°C (depending on multiplex; Table 2) for 30 s, and an extension step at 72°C for 1 min. A final extension at 72°C for 45 min concluded the reaction. Fluorescently‐labeled amplicons were separated on an Applied Biosystems 3730xl Genetic Analyzer (Applied Biosystems), sized, and scored using GeneMapper 5.0 (Applied Biosystems), and manually verified.

**Table 2:** PCR conditions and multiplex information for 16 STR loci including primer sequences, repeat motif, fragment size in base pairs (bp), PCR conditions, GenBank accession numbers, and references

Multiplex Panel	Locus	Primer sequences (5′−3′)	Repeat motif	Fragment size (bp)	PCR conditions (primer concentrations, fluorescent dye, and annealing temperature)	GenBank accession no.	References
I	MST60	F: GGTGTGCTTGTCAGGTTTC	TG	93–97	0,05 µM, FAM, 58°C	AB001057.1	Estoup, Presa, Krieg, Vaiman, and Guyomard (1993)
		R: GTCAAGTCAGCAAGCCTCA					
	Strutta58	F: CTCGGCTCACCTCGTAATAA	AC	151–203	0,20 µM, FAM, 58°C	U60223.1	Poteaux, Bonhomme, and Berrebi (1999)
		R: GTTTCTTGAAGGACTTGAAGGACGACA^a^					
	BHMS321	F: GAAAGAGGAACCTGTCATTCC	AC	206–232	0,10 µM, FAM, 58°C	AF256743.1	Direct submission by Høyheim
		R: GTTTCTTGAAGTGTGGAGGTGATGTGAC^a^					
II	Ssa85	F: GACATTTGAGGTGGGTCCT	AC	120–126	0,10 µM, FAM, 58°C	U43692.1	Direct submission by O'Reilly et al.
		R: GTTTCTTCCGCTCCTCACTTAATCAGA^a^					
	SsaD157	F: CTATCCGGCACTAAGCAGAA	TATC	216–274	0,50 µM, FAM, 58°C	AF525204.1	King, Eackles, and Letcher (2005)
		R: GTTTCTTAGGGCTGAGAGAGGAATACAA^a^					
	Ssa412UOS	F: GATACAACACTACCATAGTACCACA	AG	100–146	0,10 µM, NED, 58°C	AJ402729.1	Cairney, Taggart, and Høyheim (2000)
		R: GTTTCTTACTCTGAGGGTGCTGAGATG^a^					
	SSsp1605	F: GATAGACGCAATGGAAGTCAG	CTAT	252–384	0,20 µM, NED, 58°C	AY081812.1	Paterson, Piertney, Knox, Gilbey, and Verspoor (2004)
		R: GTTTCTTTCTGAGGCTCCTTCTACACTG^a^					
IV	MST‐73	F: GCAGGAGGTGTGGTGTATGT	TG	110–116	0,10 µM, FAM, 58°C	AB001056.1	Estoup et al. (1993)
		R: GTTTCTTCCTAGGTGAAAAGGAAATGG^a^					
	MST‐15	F: CTCTGTGACAGGTGGATCACT	CT	199–213	0,20 µM, FAM, 58°C	AB001058.1	Estoup et al. (1993)
		R: GTTTCTTTGCAGCAGCATAATCCTCTA^a^					
	SsoSL85	F: TAGGGTTTGACCAAGGGATT	CA	103–163	0,05 µM, NED, 58°C	Z48596.1	Direct submission by Slettan et al.
		R: GTTTCTTCTTCACCACCAACAAGCATA^a^					
	OMM1152	F: GCACAATGCCTGTCCTAGAT	GA	221–289	0,10 µM, NED, 58°C	AY039634.1	Rexroad, Coleman, Hershberger, and Killefer (2002)
		R: GAACCAATCAACCAATCAGC					
V	SSsp2216	F: AACACGCAGCACAGTCAGTAT	CTAA	110–135	0,05 µM, FAM, 58°C	AY081811.1	Paterson et al. (2004)
		R: GTTTCTTCAGCATCTACACCCAGAAGAA^a^					
	Ssa197	F: GAGGCTTGTGGTGTGTGTAGT	(GT)_X_(GTGA)_X_	110–162	0,10 µM, NED, 58°C	U43694.1	Direct submission by O'Reilly et al.
		R: GTTTCTTGGCAGGGATTTGACATAACTC^a^					
	One102	F: TGGCTGTTTCAGGTATGACA	TCTA	196–200	0,10 µM, NED, 58°C	AF274518.1	Olsen, Wilson, Kretschmer, Jones, and Seeb (2000)
		R: GTTTCTTCTCTGTGGATAGGCCGATTA^a^					
VI	SsoSL438	F: ATGACAACACACAACCAAGG	AC	77–85	0,50 µM, FAM, 55°C	Z49134.1	Direct submission by Slettan et al.
		R: GTTTCTTTGGAAGCATCTGTGTTTTTG^a^					
	MST543	F: CTCCAAGGGCAACAAAACTA	CT	174–228	0,80 µM, FAM, 55°C	AB001062.1	Presa and Guyomard (1996)
		R: GTTTCTTCACAAGTCATCTGGGCATCT^a^					

a Primers that includes a tail GTTTCTT (Brownstein et al., 1996).

### 2.4 Analysis of genetic variation

Tests for linkage disequilibrium and deviations from Hardy–Weinberg equilibrium (HWE) were carried out with the software GENEPOP 4.7 (Rousset, 2008). For both HWE and linkage disequilibrium, a Markov chain method with 10,000 dememorization steps, 5,000 batches, and 10,000 iterations each was used to estimate exact *p*‐values for deficiency of heterozygotes and likelihood ratio statistics, respectively. Observed and expected heterozygosity and inbreeding coefficient for the seven river sections were calculated with GenAlEx 6.51b2 (Peakall & Smouse, 2012). Allelic richness and private allelic richness were estimated with ADZE 1.0 (Szpiech, Jacobsson, & Rosenberg, 2008) based on a standardized sample size of 16.

### 2.5 Population‐genetic differentiation

GenAlEx 6.51b2 was used to estimate pairwise population‐genetic differentiation based on *G*_ST_ (Nei & Chesser, 1983) and Jost's *D* (Jost, 2008) and to test their significance based on 9,999 random permutations. The modified false discovery rate method of Benjamini & Yekutieli (2001) was used to correct for multiple testing. Additionally, the Adegenet package (Jombart, Devillard, & Balloux, 2010) in R version 3.5.1 (R Core Team, 2018) was used for conducting a discriminant analysis of principal components (DAPC) to infer population‐genetic differentiation. The cross‐validation function was used with 100 replicates to identify the optimal number of principal components to be retained, with randomly generated training sets to avoid overfitting. The number of PCs associated with the lowest “root mean squared error” (RMSE) value was selected, and results were displayed as a scatterplot to visualize genetic differentiation of river sections.

### 2.6 Genetic structure analysis

The Bayesian clustering method implemented in STRUCTURE 2.3.4 (Pritchard, Stephens, & Donnelly, 2000) was applied to detect the presence of distinct genetic clusters and to identify individuals of potentially admixed ancestry. The admixture model with correlated allele frequencies (Falush, Stephens, & Pritchard, 2003) was run twice, once using the LocPrior option and once without. Forty replicates were conducted for each *K* from 1 to 10, with 1,000,000 MCMC steps and a burn‐in period of 100,000. The LocPrior option was chosen to assess whether additional population‐genetic structure could be detected, as it has been shown that including information on the sampling location of individuals improves clustering without leading to the detection of nonexisting population‐genetic structure (Hubisz, Falush, Stephens, & Pritchard, 2009). Additionally, we used STRUCTURE to test for hierarchical population structure, in order to assess whether higher‐level genetic structure masks fine‐scale genetic clustering. The number of genetic clusters present in the data set was estimated by four recently proposed estimators (Puechmaille, 2016): the median of means (MedMeaK), maximum of means (MaxMeaK), median of medians (MedMedK), and maximum of medians (MaxMedK), using the program STRUCTURESELECTOR (Li & Liu, 2018) to account for uneven sample sizes in the data set. The program CLUMPAK (Kopelman, Mayzel, Jakobsson, Rosenberg, & Mayrose, 2015) was used to visually summarize results from the separate STRUCTURE runs. Finally, significance of differences in observed heterozygosity between the genetic clusters was tested with FSTAT 2.9.3.2, based on 5,000 permutations (Goudet, 2002).

### 2.7 Bottleneck analysis

To test for recent reductions in effective population sizes (i.e., genetic bottlenecks), the program BOTTLENECK 1.2.02 (Piry, Luikart, & Cornuet, 1999) was used. The algorithm in BOTTLENECK assumes that allelic diversity is lost at a faster rate than heterozygosity and, therefore, tests for an excess of heterozygosity compared to expectations at mutation‐drift equilibrium (Cornuet & Luikart, 1996). Following recommendations by Peery et al. (2012), two mutation models were assessed, the infinite‐allele model (IAM) and the two‐phase model (TPM). The TPM model allows different proportions of microsatellites to follow either the IAM or the stepwise mutation model (SMM) and so the model was run three times for each population, assuming that the percentage of stepwise mutations was 20%, 50%, and 70%, respectively. The 1‐way Wilcoxon sign‐rank test (Luikart, 1997) was applied to assess significance.

## 3 RESULTS

### 3.1 Genetic variation

The result of the microsatellite optimization was five novel multiplexes consisting of 16 STR markers (Table 2). None of the markers showed signs of null alleles, large allele dropout, or scoring errors, so all loci were retained for further analysis. Equally, none of the tests for Hardy–Weinberg equilibrium or linkage disequilibrium were statistically significant after Bonferroni correction (heterozygosity deficit: 11/126; linkage disequilibrium: 37/995 were significant before correction). Nonstocked Russian sections showed lower observed and expected heterozygosity values than did the stocked Norwegian–Russian sections, with the exception of section E (Table 3). Consistent with this finding, combined Russian sections A–C and Norwegian–Russian sections D–J showed significant differences in observed and expected heterozygosity (*p* = 0.048). Private allelic richness was lowest in the Norwegian–Russian sections G, H, and I, with section F having the highest private allelic richness. In addition, section H (Melkefoss) showed a negative inbreeding coefficient, possibly indicating outbreeding.

**Table 3:** Genetic summary statistics

	*N*	*H*_O_ (*SE*)	*H*_E_ (*SE*)	*F*_IS_ (*SE*)	*A*_R_	*A*_PR_
Zone B	32	0.586 (0.06)	0.579 (0.06)	−0.005 (0.02)	4.245	0.265
Zone C	20	0.547 (0.05)	0.596 (0.05)	0.062 (0.03)	4.463	0.245
Zone E	12	0.594 (0.07)	0.591 (0.05)	0.031 (0.07)	4.335	0.190
Zone F	8	0.622 (0.05)	0.614 (0.04)	−0.009 (0.05)	4.511	0.392
Zone G	51	0.638 (0.06)	0.636 (0.05)	0.004 (0.03)	4.414	0.112
Zone H	25	0.673 (0.05)	0.637 (0.05)	−0.075 (0.04)	4.421	0.214
Zone I	22	0.621 (0.04)	0.644 (0.05)	0.024 (0.03)	4.541	0.228

Abbreviations: *A*_PR_, private allelic richness; *A*_R_, allelic richness; *F*_IS_ (*SE*), inbreeding coefficients with standard error; *H*_E_ (*SE*), expected heterozygosity with standard error; *H*_O_ (*SE*), observed heterozygosity with standard error; *N*, number of individuals.

### 3.2 Population‐genetic differentiation

Estimates of population‐genetic differentiation (*G*_ST_ and Jost's *D*, Table 4a,b, respectively) across river sections consistently showed that nonstocked Russian sections were significantly genetically differentiated from each other and from stocked Norwegian–Russian sections, except for the pairwise comparison involving sections C and F. In the Norwegian–Russian sections, pairwise genetic differentiation values were mostly nonsignificant for both estimators, indicating a lack of population‐genetic substructuring. The only exceptions found here were two pairwise values (E–G and G–I) that were significant after correcting for multiple tests.

**Table 4:** Pairwise genetic differentiation (*G*_ST_, [a]; Jost's *D*_EST_, [b]) between river sections (below diagonal) and respective *p* values (above diagonal)

	B	C	E	F	G	H	I
(a)							
B	–	**0.001**	**0.000**	**0.000**	**0.000**	**0.000**	**0.000**
C	0.009	–	**0.003**	0.122	**0.000**	**0.000**	**0.000**
E	0.031	0.013	–	0.404	**0.003**	0.015	0.020
F	0.034	0.006	0.001	–	0.127	0.041	0.137
G	0.035	0.021	0.011	0.005	–	0.201	**0.001**
H	0.036	0.025	0.008	0.008	0.001	–	0.713
I	0.036	0.020	0.009	0.006	0.007	−0.001	–
(b)							
B	–	**0.001**	**0.000**	**0.000**	**0.000**	**0.000**	**0.000**
C	0.029	–	**0.002**	0.113	**0.000**	**0.000**	**0.000**
E	0.096	0.041	–	0.403	**0.003**	0.017	0.023
F	0.117	0.021	0.003	–	0.122	0.039	0.136
G	0.116	0.071	0.038	0.019	–	0.200	**0.001**
H	0.123	0.088	0.026	0.031	0.005	–	0.713
I	0.126	0.073	0.031	0.022	0.028	−0.005	–

Note

Significant *p* values after a modified false discovery rate correction for multiple tests (critical level *p* = 0.014) are highlighted in bold.

**Figure 3:**
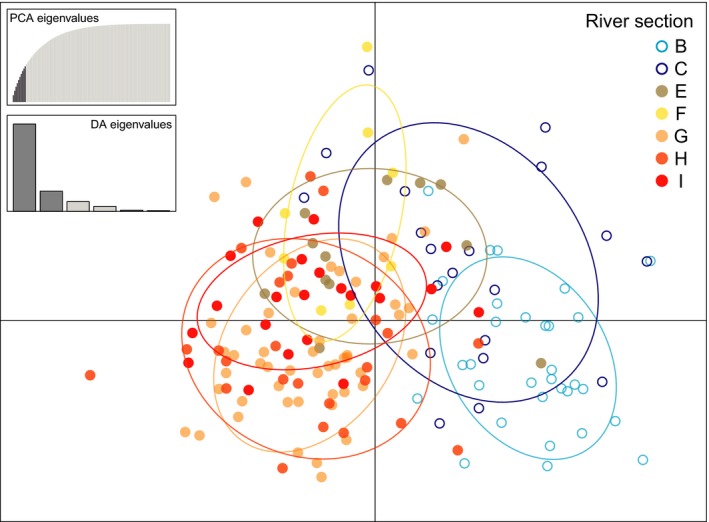
Discriminant analysis of principal components (DAPC) ordination of 170 Pasvik River brown trout individuals based on data from 16 STR loci. Letters correspond to river sections given in Figure 2. The left insets display the PCA eigenvalues and the DA eigenvalues in relative magnitude

The DAPC ordination (Figure 3) was consistent with the population‐genetic differentiation estimates in that a clear separation between nonstocked Russian sections B and C and stocked Norwegian–Russian sections G–I is visible. Further, the genetic distance between sections B and C is larger than among Norwegian–Russian sections, with the exception of sections E and F, which are situated in the center of the scatterplot (Figure 3).

### 3.3 Genetic structure results

**Figure 4:**
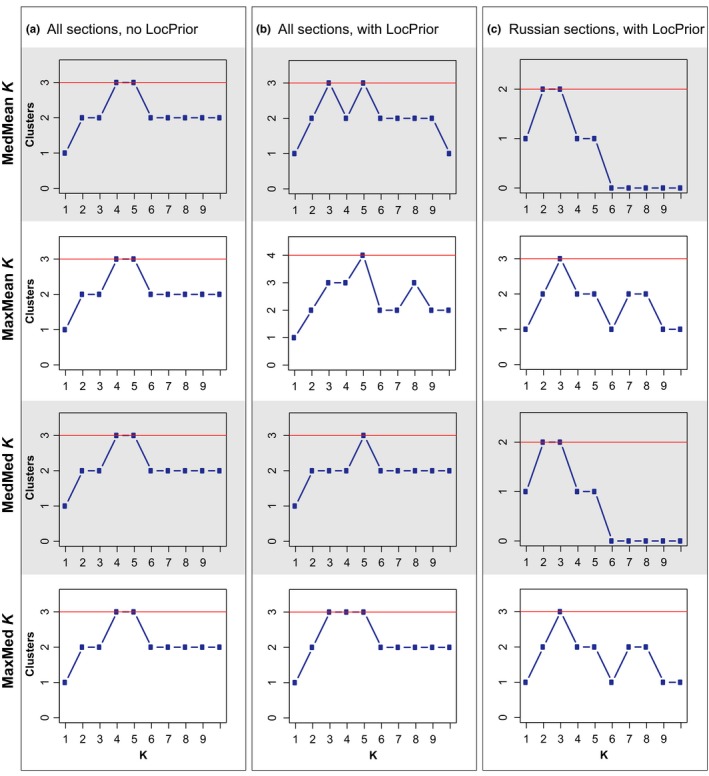
Estimation of the most likely number of genetic clusters (=*K*) present in the data set. The three columns (a–c) show the likely number of genetic clusters present for the three different STRUCTURE runs (i.e., all sections without LocPrior, all sections with LocPrior, and Russia with LocPrior, respectively) as estimated by four estimators: the median of means (MedMeaK), maximum of means (MaxMeaK), median of medians (MedMedK), and maximum of medians (MaxMedK; Puechmaille, 2016)

**Figure 5:**
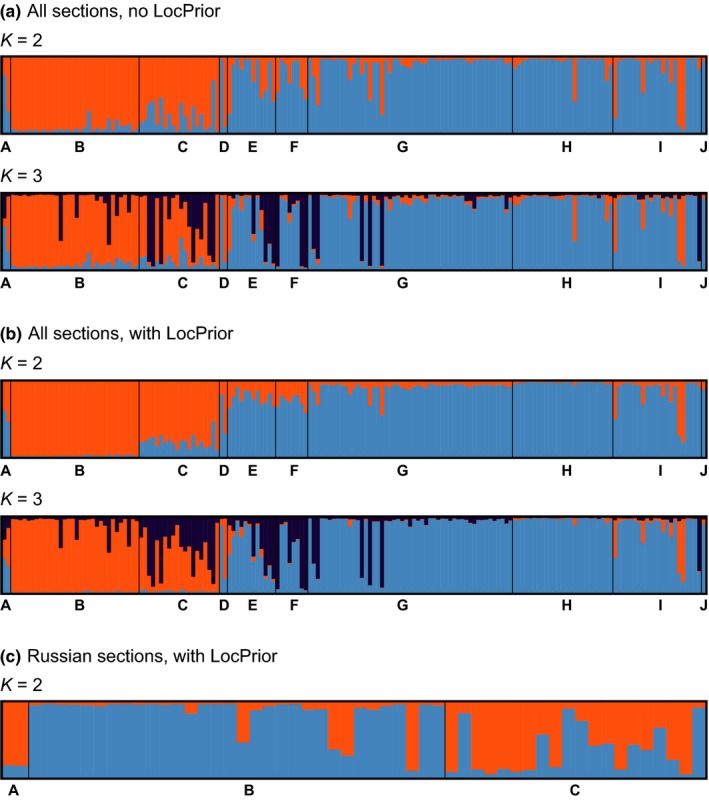
STRUCTURE bar plots. (a) STRUCTURE bar plots for *K* = 2 to *K* = 3 for all river sections (not using LocPrior). (b) STRUCTURE bar plots for *K* = 2 to *K* = 3 for all river sections (using LocPrior). (c) STRUCTURE bar plots for *K* = 2 for the upper Russian part (using LocPrior)

The different estimators in STRUCTURESELECTOR provided support for 3–4 genetic clusters in the whole data set (Figure 4a–c) regardless of whether the LocPrior option was used or not. Nevertheless, the posterior LocPrior parameter (mean *r* = 1.64 for *K* = 3) indicated that location information was fairly informative for assisting in genetic clustering. At *K* = 3, the three groups generally corresponded to the nonstocked river sections A–C in the Russian part and to the stocked sections G–J in the Norwegian–Russian part, while sections in‐between (i.e., E and F) partially showed assignment to a third genetic cluster (Figure 5a,b). A few individuals in section G showed assignment to this third cluster as well. Although less supported, it is noteworthy that at *K* = 2, genetic structuring essentially separated the nonstocked Russian sections from stocked sections in the Norwegian–Russian part of the river. Individuals that were assigned to the third cluster at *K* = 3 were mostly unassigned at *K* = 2, and were concentrated in the area connecting the stocked and nonstocked river sections. Additional STRUCTURE runs in which 25 individuals were randomly selected from sections B and G to reduce unevenness in the data set in terms of sample sizes verified the presence of the main genetic clustering described above as well as the main admixture patterns present in the center of the river (results not shown). Testing for hierarchical genetic structure within the two main clusters revealed that two sections (B and C) in the nonstocked Russian part of the river corresponded to two subtle genetic clusters. This was consistent with the population‐genetic differentiation results, indicating significant genetic structuring of adjacent river sections caused by a single dam (Figure 5c). No further substructure was found within the stocked Norwegian–Russian part with an additional STRUCTURE run using only samples from sections D–J (results not shown).

### 3.4 Demographic history

Bottleneck tests carried out to test for recent reductions in effective population size revealed that stocked Norwegian–Russian sections G, H, and I likely have undergone recent bottlenecks, as demonstrated by statistically significant heterozygosity excess under both the infinite‐allele model and the two‐phase mutation model (Table 5). No bottleneck signatures were found in the nonstocked Russian sections or in Norwegian–Russian sections E and F, which show higher admixture with the Russian part and also potentially ancestry to a separate third genetic cluster.

**Table 5:** Tests for genetic bottlenecks using BOTTLENECK

	IAM	TPM_70	TPM_50	TPM_20
B	**0.047**	0.530	0.281	0.174
C	0.080	0.334	0.202	0.137
E	0.088	0.490	0.372	0.188
F	0.188	0.702	0.647	0.470
G	**0.001**	**0.033**	**0.009**	**0.003**
H	**0.009**	**0.037**	**0.017**	**0.011**
I	**0.003**	**0.012**	**0.005**	**0.004**

Note

Tests were performed using the infinite‐allele model and the two‐phase mutation model (TPM). For the latter, different proportions of STR loci that follow the stepwise mutation model were used (i.e., 70%, 50%, and 20%, respectively). *p*‐values according to a 1‐way Wilcoxon rank test for heterozygote excess are given, and significant values are highlighted in bold font.

## 4 DISCUSSION

The current study provides evidence that patterns of fine‐scale genetic diversity and differentiation are governed by both hydroelectric dams and restocking in a transnational subarctic riverine system. In nonstocked parts of the Pasvik River, dams contributed to significant genetic differentiation between adjacent river sections, whereas this effect was absent in the stocked parts. Additionally, heterozygosity was comparatively low in nonstocked compared to stocked river sections. However, in the stocked river sections G–I, reduced levels of private allelic richness and signals of recent bottlenecks could be detected. In essence, the artificial dispersal barriers created by the dams, in combination with long‐term stocking, have chiefly impacted the genetic diversity and contemporary patterns of genetic differentiation of brown trout in the watercourse. Furthermore, genetic signs of bottlenecks in some of the stocked sections provide evidence of genetic swamping of the wild trout population by interbreeding with stocked, hatchery‐reared fish.

### 4.1 Effects of stocking on genetic diversity and demography

Brown trout subpopulations in the Norwegian–Russian sections of the Pasvik River generally showed elevated observed and expected heterozygosity values, and section H showed signals of outbreeding as indicated by a negative inbreeding coefficient. At the same time, bottleneck tests showed that sections G, H, and I in the stocked sections have experienced a recent bottleneck event, pointing to a reduced breeding population size. Thus, the elevated heterozygosity values may be interpreted as a sign of high genetic diversity, while the bottleneck results point to a reduction in genetic diversity. These apparently contradictory results may be explained by the fact that allelic diversity is lost at a faster rate than heterozygosity in diminishing populations (Piry et al., 1999), which allows the detection of bottlenecks despite fairly high heterozygosity in the respective population. For some genetic metrics, like heterozygosity a time lag for disturbance events to manifest themselves in those metrics has been reported (Landguth et al., 2010; Piry et al., 1999). However, bottleneck detection is also dependent on the mutation model, number of loci, and sample size used (Peery et al., 2012) and violations of assumptions may lead to erroneous detection of bottleneck signals in stable populations. Here, a bottleneck signal was only interpreted to be biologically meaningful if significance was achieved in all four tests (Table 5), thereby considerably reducing the probability of false positives. Further, our set of 16 STRs was substantially larger than the median number of 8–9 loci reported by Peery et al. (2012). Finally, sample sizes were either higher or close to the averages given in Peery et al. (2012). Overall, this indicates that most recommendations for bottleneck tests by Peery et al. (2012) were met.

Theoretical and simulation studies (Morrissey & de Kerckhove, 2009; Paz‐Vinas & Blanchet, 2015; Paz‐Vinas, Loot, Stevens, & Blanchet, 2015) predict that population sizes and genetic diversity might be naturally higher in downstream than in upstream river sections. Two main processes have been proposed to explain this prediction: (a) downstream‐biased gene flow caused by asymmetric dispersal costs in unidirectional water flow (Morrissey & de Kerckhove, 2009) and (b) variation in habitat availability, with larger habitat areas usually being available downstream due to increased river width (Carrara, Rinaldo, Giometto, & Altermatt, 2014). Generally, it is more likely that potential higher genetic diversity results from more variation in habitat availability in the current study because downstream‐biased gene flow is low or absent because of the dams. The prediction of higher genetic diversity in downstream river sections may be partially supported by this study based on relatively high heterozygosity values found downstream. However, this commonly used measure for genetic diversity may be misleading in this case because stocking results in the release of a high number of offspring from relatively few parents, leading to a bottleneck signal indicating that genetic diversity is being lost at the same time. Thus, theoretical predictions about spatial distribution of genetic diversity may be considerably altered by anthropogenic changes, and this needs to be taken into account when using genetic diversity measures to inform management.

### 4.2 Effects of stocking on population‐genetic differentiation

The most likely partitioning scheme resulting from the Bayesian STRUCTURE analysis divided the analyzed brown trout individuals into three genetic clusters. The first two clusters essentially correspond to the nonstocked Russian (A–C) and the stocked Norwegian–Russian (G–J) sections of the river, while the third cluster is most common in the center of the river system (sections C–F). At *K* = 3, two genetic clusters essentially delimited nonstocked from stocked river sections. The location of the main subdivisions suggests that stocking in the Norwegian–Russian part has led to genetic differentiation from the nonstocked Russian sections of the river, and that the gene pools of the most heavily stocked sections of the river (G, H, and I) have essentially been homogenized by the annual release of several thousand hatchery‐bred individuals. Stocking has been shown to contribute to substantial genetic differentiation and drift effects in salmonids, leading to rapid divergence of wild and captivity‐bred fish (Hansen et al., 2009; Laikre et al., 2010; Quiñones et al., 2014). Similarly, stocking probably provides an explanation for the genetic differentiation between stocked and nonstocked river sections in our study. However, the origin of the third genetic cluster is less clear. Looking at the *K* = 3 STRUCTURE bar plot (Figure 5a,b), one possibility is that admixture of individuals from stocked and nonstocked parts or the river led to an additional genetically differentiated cluster over time in the center of the watercourse. We cannot rule out that occasional downstream migration of individuals through dams contributes to this pattern. An alternative explanation is that flooding led to a carry‐over of fish specimens in this river section. Both migration and flooding also allow for the introduction of genetically different individuals from side rivers and hence the introduction of new genetic material. In connection to this, sections E and F, including their side rivers, are assumed to have larger intact spawning sites and nursery areas for brown trout, probably allowing for the retention of natural genetic diversity.

Despite the existence of four hydroelectric dams, genetic differentiation was generally low among sections of the Norwegian–Russian part of the Pasvik River. Within this stocked part, differentiation was statistically significant only for two pairwise comparisons involving section G. This particular section is short and therefore possibly has a lower population size than other river sections. It also shows the lowest private allelic richness, suggesting that the genetic differentiation in this case is caused by drift. Finally, although stocking in section G was stopped more than a decade ago, it is possible that past extensive stocking introduced alleles that led to the significant genetic differentiation. The general genetic uniformity of the brown trout population of the Norwegian–Russian river part indicates that stocking has homogenized the gene pools of the separate river sections. Contrastingly, in the upstream Russian part of the river, sections B and C showed significant genetic differentiation and were identified as distinct genetic clusters in the STRUCTURE analysis. Hence, in these nonstocked parts of the river, dams contributed to genetic differentiation. This finding is in line with previous studies in brown trout and other salmonids showing that artificial barriers can quickly lead to isolation and genetic differentiation (Heggenes & Røed, 2006; Meldgaard et al., 2003; Stelkens et al., 2012).

Paradoxically, our results suggest that the three hydroelectric dams of the headwater sections of Pasvik River may protect wild trout populations in Russia from genetic swamping by hatchery‐reared fish of Norwegian–Russian origin by preventing their upstream migration. Indeed, several studies have pointed out that removal of barriers or the installment of fish passes could have unintended detrimental consequences for unique genetic diversity in natural populations of brown trout and other aquatic species (Baric et al., 2010; Van Houdt et al., 2005; Rahel, 2013). Due to widespread anthropogenic habitat destruction across the distribution range of the Eurasian brown trout, as well as the long‐term practice of translocations and stocking, natural and genetically unaltered trout populations are becoming increasingly rare (Araguas et al., 2009, 2017; Baric et al., 2010). Consequently, the identification and preservation of populations with high genetic integrity is a primary long‐term conservation goal in brown trout (Araguas et al., 2009, 2017; Baric et al., 2010). The Pasvik River is one of the largest and most species‐rich subarctic river systems in northwestern Eurasia, with partially remaining wild brown trout populations; conservation of these natural populations is important to preserve unique genetic diversity in the region. Although brown trout as a species is not endangered, harvest, translocations, and stocking have heavily altered natural populations, and it is therefore crucial to preserve remaining wild populations in order to retain the full spectrum of intraspecific genetic and ecological diversity (Berrebi et al., 2019; Piccolo et al., 2017).

To conclude, our results indicate that stocking alleviated the negative genetic effects of habitat fragmentation caused by dams by reducing genetic differentiation, but also that stocking is the likely cause of genetic diversity loss, as demonstrated by the significant bottleneck results. This suggests that the stocking program in the Pasvik riverine system would benefit from a larger parental breeding pool, in order to prevent further genetic diversity loss and to ensure long‐term population viability. Likewise, increasing natural recruitment by restoration of spawning areas may be a viable option to support a larger breeding pool. In a broader context, the present study points to the fact that contrasting management strategies across international borders were insufficient to address certain sustainable management goals that aim for the long‐term protection of natural genetic diversity, connectivity, and evolutionary potential. Consequently, our results suggest that transnational harmonization of mitigation strategies based on more research may be warranted to develop efficient large‐scale conservation management strategies that link local genetic diversity to large‐scale genetic integrity for native brown trout in the Pasvik riverine system and across Eurasia.

## CONFLICT OF INTEREST

None declared.

## AUTHOR CONTRIBUTIONS

SH conceptualized and designed the study and wrote first draft together with CFCK. CFCK additionally analyzed the data. KF carried out laboratory analyses. NP contributed samples from Russia. All authors commented, interpreted, and discussed the results and contributed critical feedback for the final version of the study, including analytical steps and background information. PEA provided background information on ecosystem specifics. All authors approved the final version of the article.

## DATA ACCESSIBILITY

The microsatellite data set has been deposited in the Dryad Digital Repository (DOI: https://doi.org/10.5061/dryad.45482t5).
